# Pleomorphic liposarcoma of bone: a rare primary malignant bone tumour

**DOI:** 10.1186/s13569-018-0089-7

**Published:** 2018-02-09

**Authors:** G. L. Tiemeier, J. M. Brown, S. E. Pratap, C. McCarthy, A. Kastrenopoulou, K. Bradley, S. Wilson, Z. Orosz, C. L. M. H. Gibbons, U. Oppermann, N. A. Athanasou

**Affiliations:** 1 Nuffield Department of Orthopaedics, Rheumatology and Musculoskeletal Sciences, Nuffield Orthopaedic Centre, University of Oxford, Oxford, OX3 7HE UK; 2 Sarcoma Service, Nuffield Orthopaedic Centre, Oxford, UK; 3 Department of Radiology, Nuffield Orthopaedic Centre, Oxford, UK; 4 Department of Radiology, Churchill Hospital, Oxford, UK

**Keywords:** Liposarcoma, Bone, Malignant, Primary tumour

## Abstract

**Background:**

Liposarcoma is an extremely rare primary bone sarcoma.

**Case presentation:**

We report a case of primary pleomorphic liposarcoma that arose in an 18 year old male in the metaphysis of the left tibia. Plain radiographs showed a partly sclerotic lesion and MR imaging a heterogeneous tumour predominantly isointense on T1- and high-signal on T2-weighted sequences with focal areas of increased T1 signal that suppressed with fat saturation. PET/CT showed marked FDG uptake (SUV = 17.1) in the primary tumour as well as a metastasis in the right distal femur and multiple small pulmonary metastases. Histologically, the tumour was a pleomorphic liposarcoma containing large tumour cells with vacuolated cytoplasm and hyperchromatic pleomorphic nuclei as well as numerous lipoblasts and scattered brown fat-like cells. Tumour cells strongly expressed FABP4/aP2, a marker of adipocyte differentiation, and UCP1, a marker of brown fat, but not S100. The case was treated with neoadjuvant MAP chemotherapy, resulting in extensive (> 95%) necrosis in the primary tumour and almost complete resolution of the femoral and pulmonary metastases.

**Conclusions:**

Pleomorphic liposarcoma can present as a sclerotic primary malignant bone tumour; markers of adipose differentiation are useful in histological diagnosis and neoadjuvant MAP chemotherapy results in significant tumor necrosis.

## Background

Liposarcoma is an extremely rare primary malignant bone tumour defined in the 2005 AFIP Fascicle as “A malignant tumor with differentiation towards adipocytes” [1]. Catto and Stevens [2], reviewing the world literature in 1963, could find only 15 cases of primary bone liposarcoma; they noted that most reported cases were pleomorphic sarcomas and considered only one case, that reported by Dawson in 1955 [3], as completely convincing. In 1982 Addison and Payne accepted only six examples of this tumour in previously published reports [4]. Subsequently, other cases have been reported, but there is considerable uncertainty regarding the histological diagnosis and optimum treatment of this rare tumour.

We report the clinical, radiological (including MRI and PET/CT) and pathological findings in a case of primary pleomorphic liposarcoma that arose in the proximal tibia of an 18 year old male. We also review the literature regarding primary liposarcoma of bone and reassess diagnostic criteria and treatment options in the light of our findings.

## Case presentation

An 18 year old white male presented with a 6 month history of discomfort and swelling in his left knee. An X-ray and MRI of the left knee revealed a large, partly mineralized tumour in the medial tibial diaphysis. In his past history, the patient had been diagnosed at birth with coarctation of the aorta and primary lymphoedema. Balloon dilatation angioplasty was successfully performed on day 13 of life. Subsequent cardiac medical management included nifedipine between the ages of 4 and 9; this was later switched to atenolol because of lymphoedema. His phenotype was consistent with a variant of Irons–Bianchi syndrome or ‘‘Milroy-like’’ lymphoedema. Further details of his medical history and treatment are described elsewhere [5]. Genetic testing to date has proven negative for pathogenic variants, including the “Red Cell gene panel” and the 23 genes in the “Rasopathy panel” although heterozygous variations of uncertain clinical significance were identified in the FAT4 gene (c.8290A > C and c.12070C > T). He had also received topical bleomycin and acitretin for persistent plantar viral warts. The patient had no family history of congenital syndromes, but there was a strong family history of cancer including renal cell carcinoma, skin cancer, bowel cancer and breast cancer in second-degree relatives.

### Radiological findings

**Fig. 1:**
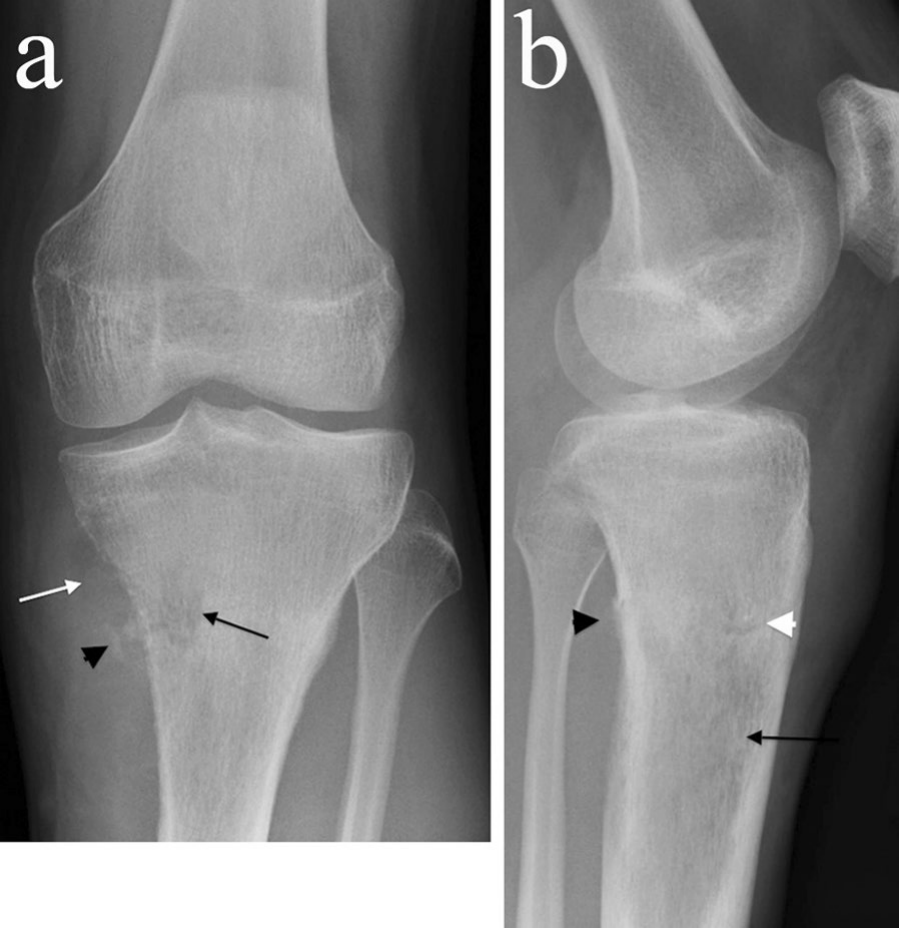
**a** AP and lateral **b** plain radiographs demonstrate an ill-defined radiolucent lesion in the proximal tibial metadiaphysis (black arrows) with posteromedial cortical destruction and adjacent ossification (black arrowheads). A medial soft tissue mass with low density raises the possibility of a fatty matrix (white arrow). A pathological fracture is present (white arrowhead)

Plain radiographs demonstrated a poorly defined, permeative, radiolucent lesion, centred in the proximal tibial meta-diaphysis. A pathological fracture was present (Fig. 1).

There was postero-medial cortical destruction with adjacent areas of ossification. There was evidence of low density in the medial soft tissue mass, raising the possibility of a fatty matrix.

**Fig. 2:**
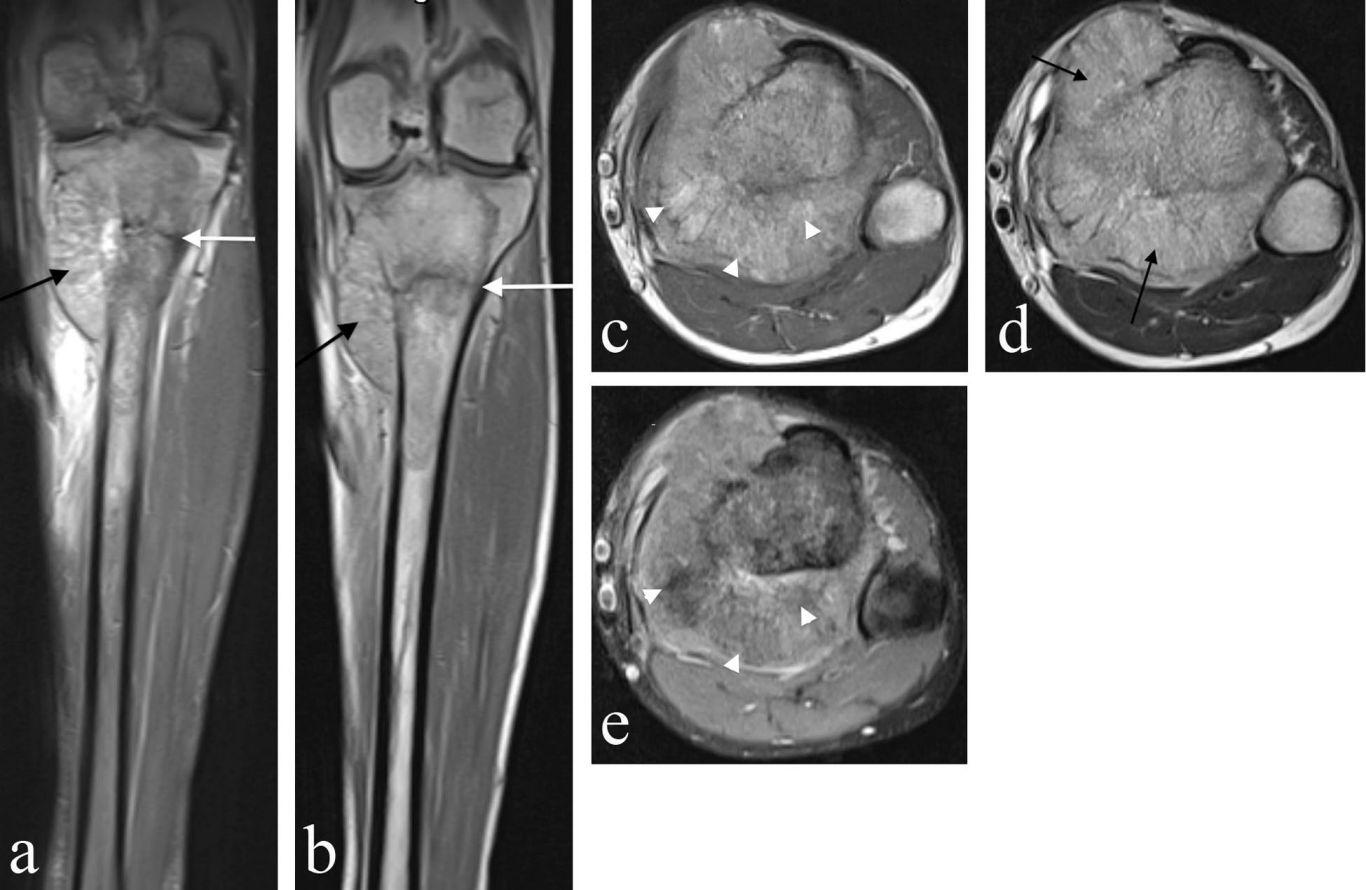
Magnetic Resonance Imaging (MRI) of the proximal tibial lesion. **a** Coronal STIR, **b** coronal T1-, **c** axial T1-, **d** axial T2-weighted and, **e** axial proton density with fat saturation. The images demonstrate an ill-defined proximal tibial medullary based lesion extending to the subarticular surface. There is extensive cortical destruction with an almost circumferential soft tissue mass, which has a spiculated appearance (black arrows) displacing the posterior muscles and popliteal neurovascular bundle. Tumour breaches the deep fascia anteriorly to extend into the subcutaneous tissues and penetrates the interosseous membrane to extend around the anterolateral tibial cortex. Heterogeneous partly high T1 signal suppresses with fat saturation sequences supporting a partly fatty matrix (white arrowheads). The pathological fracture is clearly seen extending transversely across the proximal tibia (white arrows)

Magnetic resonance imaging (MRI) showed that the tumour filled the medullary cavity of the proximal tibial metaphysis; it had an ill-defined margin and crossed the physis extending to the subarticular surface but there was no intra-articular extension (Fig. 2). Extensive medial cortical destruction was associated with an almost circumferential spiculated soft tissue mass which had displaced the calf musculature and popliteal neurovascular bundle. The tumour breached the deep fascia and the interosseous membrane. The lesion was of heterogeneous isointense signal on T1- and high signal on T2-weighted images. Areas of high T1 signal suppressed on fat saturation sequences, supporting a partly fatty matrix. A pathological fracture was clearly seen extending transversely across the proximal tibia.

**Fig. 3:**
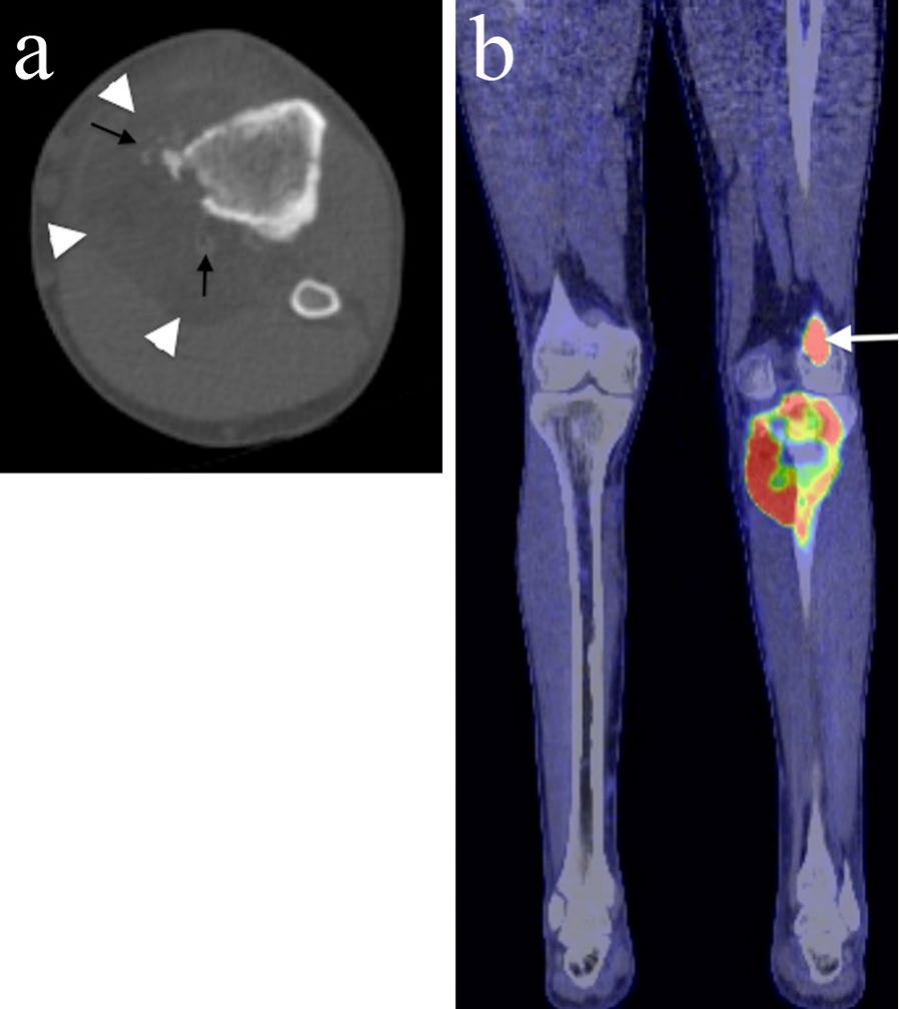
Axial CT and PET/CT imaging of the proximal tibial lesion. **a** Axial CT imaging shows medial cortical destruction and a large soft tissue mass containing areas of fat attenuation (white arrowheads) and ossification (black arrows). **b** A coronal fused PET/CT image shows there is marked FDG uptake in the proximal tibial tumour as well as in a lateral distal femoral metastasis (white arrow). No other lipomatous lesion or tumour is present

Positron emission tomography with fluorodeoxyglucose (FDG) integrated with computed tomography (PET/CT) confirmed an ill-defined proximal tibial lesion with medial cortical destruction and a large soft tissue mass containing areas of fat attenuation and ossification (Fig. 3a). The tumour showed marked FDG uptake with a standardised uptake value (SUV) of 17.1 and a band of relative photopaenia in the region of the undisplaced transverse pathological fracture. A further focus of markedly increased FDG uptake with an SUV of 13.5 was seen in a lateral distal femoral lesion which was presumed to represent a metastasis (Fig. 3b). Eight small bilateral pulmonary nodules (maximum 4 mm) were also noted on the PET/CT scan consistent with lung metastases. There was no lymphadenopathy, and no fat or other soft tissue lesion was noted.

### Histological findings

**Fig. 4:**
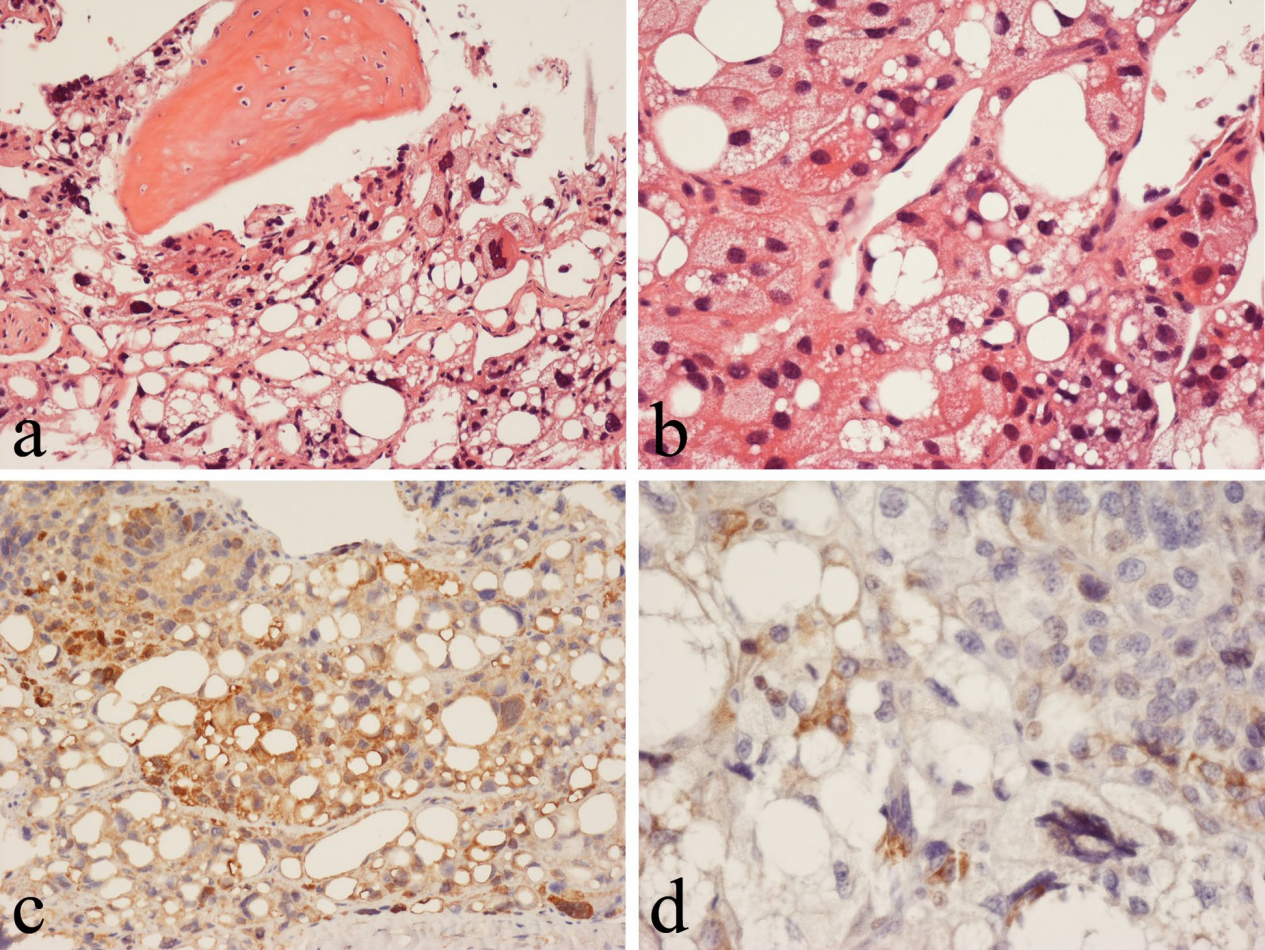
Histological analysis of the proximal tibial lesion shows primary pleomorphic liposarcoma of bone. **a** The tumor infiltrates cancellous bone and is composed of numerous lipoblasts and large pleomorphic cells, which have vacuolated cytoplasm and atypical nuclei. **b** Lipoblasts and brown fat-like cells are seen within the tumour. Immunohistochemistry shows the tumor cells express FABP4/aP2 (**c**) and UCP1 (**d**)

Histology of a biopsy of the tibial mass revealed a proliferation of malignant cells that had vacuolated cytoplasm and large atypical pleomorphic nuclei (Fig. 4). There were vacuolated tumour giant cells and numerous small lipoblast-like cells with a single cytoplasmic fat vacuole and hyperchromatic nuclei as well as brown fat-like cells with multiple small fat vacuoles (Fig. 4a, b). There were frequent mitotic figures, many of which were atypical. No evidence of osteoid formation was seen. There was infiltration of cancellous bone and evidence of lymphovascular invasion. Immunohistological analysis showed that the malignant cells strongly expressed FABP4/aP2 [6, 7], a marker of adipocyte differentiation and UCP1 [8–10], a marker of brown adipose tissue (Fig. 4c, d); there was no expression of S100, desmin, smooth muscle/muscle actin, myogenin, CD34, CD31, CD30, CD45, cytokeratin, epithelial membrane antigen, CD99 or CD117. Cytogenetic analysis showed that there was no evidence of MDM2 or CDK4 amplification. The morphological and immunohistochemical features were thought to be most in keeping with a diagnosis of primary pleomorphic liposarcoma of bone.

### Oncological and surgical treatment

The patient was treated with two cycles of neoadjuvant multi-agent chemotherapy, comprising methotrexate, doxorubicin and cisplatin (MAP), as per the closed European American Osteosarcoma (EURAMOS) trial. His baseline pre-treatment 2D transthoracic echocardiogram was within normal limits with an ejection fraction of 70%. The patient tolerated the chemotherapy well with clinical improvement after 2 cycles of chemotherapy as evidenced by a significant reduction in his analgesia use. The preoperative MRI showed an interval reduction of the large primary left tibial lesion but an increase in size and interval of the right distal femoral lesion. The preoperative PET scan demonstrated a discordant excellent metabolic response to neoadjuvant chemotherapy within both the primary tumour and the metastatic lesions. The FDG uptake was significantly reduced in the primary tumour of the tibia from 17.1 to 2.7 and the focus in the femur and right tibia showed only background activity. The majority of the bilateral pulmonary nodules had also resolved.

**Fig. 5:**
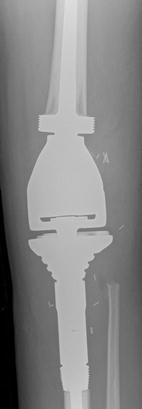
Post-resection AP plain radiograph demonstrates a Stanmore prosthesis with integral distal femur and modular proximal tibial component

The patient underwent an uncomplicated surgical resection of the primary tumour in the proximal tibia and the lesion in the distal femur with reconstruction using a linked distal femur and proximal tibial endoprosthesis (Stanmore Implants, UK) (Fig. 5). At operation, the left proximal tibial tumour was noted to be yellow and necrotic; it filled the medullary cavity and had spread through the bone cortex into covering soft tissues. A second yellow nodule was present in the left distal femur. Histologically, the resected tibia contained only a small amount of residual viable tumor that was similar morphologically and immunohistochemically to that seen in the biopsy. There was extensive (> 95%) tumor necrosis as a consequence of neoadjuvant chemotherapy.

Post-operative adjuvant chemotherapy was considered appropriate in this case because of the excellent response to chemotherapy. As routine cardiac assessment demonstrated an asymptomatic significant reduction in ejection fraction (70–53%), adjuvant chemotherapy was changed to 5 cycles of ifosfamide and etoposide, because of the previously reported activity of ifosfamide in liposarcoma [11]. 12 months after surgical excision of the tibial and femoral lesions, the patient is well with no evidence of metastasis or recurrence on clinical and radiological (including PET scan) follow up.

## Discussion

Although liposarcoma is a relatively common malignant soft tissue tumour, it has only rarely been reported in bone (Table 1). In most of these reports, the tumour was simply described as a primary bone sarcoma containing numerous vacuolated pleomorphic tumour cells with the tumour considered to represent a liposarcoma mainly on cytomorphological grounds [2–4, 12–29]. Several of these reports have been questioned on the basis of histological interpretation and uncertainty as to whether the lesion was entirely located within bone. In more recent reports, five such tumours have been specifically designated as pleomorphic liposarcomas [30–34]. In the majority of reported cases, liposarcoma of bone has been noted to develop in major long tubular bones including femur, tibia and humerus with most cases located in the lower limb. The tumour has been reported to occur over a wide age range (13–69 years), with an average age of 38 years; only six of the cases previously reported occurred in patients under the age of 25 years. In our case, the tumour arose in the proximal tibia of an 18 year old male.

**Table 1:** Reported cases of primary liposarcoma of bone

Authors (reference)	Age/sex	Site	Subtype	Treatment	Metastasis	Outcome (comment)
Fender [12]	23/F	Fibula?	Unknown	Decompression	Intracranial	Alive, 22 months follow-up (primary uncertain)
Barnard [13]	30/F	Humerus	Unknown	Amputation	Lung	Died after 2 months
Rehbock et al. [14]	56/F 60/M	Femur Iliac bone	Unknown Unknown	Stabilisation and radiation –	Bone, lymph nodes –	Died after 14 months (case not convincing) Died after 2 weeks (case not convincing)
Duffy et al. [15]	49/M	Femur	Unknown	Amputation, radiation	–	Alive, 60 months follow-up
Dawson et al. [3]	28/F	Femur	Unknown	Amputation	Lung	Died after 11 months
Retz [16]	40/M	Tibia	Unknown	Amputation	–	Alive 24 months follow-up
Johnson et al. [17]	25/M 46/M	Humerus Humerus	Unknown Unknown	Amputation –	Lung Lung	Died after 26 months Died after 18 months
Catto et al. [2]	16/F	Tibia	Unknown	Amputation	Lung	Alive, 9 months follow-up
Goldman et al. [18]	33/M	Ulna	Unknown	Amputation	–	Alive, 5 months follow-up
Ross et al. [19]	15/M	Fibula	Unknown	Resection, radiation	Lung	Died after 5 months
Schwartz et al. [20]	49/M	Tibia	Unknown	Amputation	–	Alive, 7 months follow-up
Larsson et al. [21]	52/F	Femur	Unknown	Radiation	Lung	Died after 5 months
Schneider et al. [22]	69/M	Fibula	Unknown	Amputation	–	Unknown, 24 months follow-up
Pardo-Mindan et al. [23]	39/M	Humerus	Unknown	Unknown	Unknown	Unknown
Cremer et al. [24]	58/F 37/F	Femur Ilium	Unknown Unknown	Amputation Radiation, methotrexate, cordotomy	Lung Cerebral	Progression after 2,5 years with pulmonary metastasis Died after 3 years of cerebral metastasis
Downey et al. [25]	15/F	Ischium Ilium	Unknown	Hemi-pelvectomy	Lung	Died after 2 months of pneumothorax, Pleomorphic liposarcoma with osteosarcomatous foci
Addison et al. [4]	19/M	Humerus	Unknown	Amputation, radiation, chemotherapy	Lung	Died after 10 months
Kenan et al. [26]	57/M	Scapula	Myxoid	Curettage	–	Alive, 36 months follow up
Seo et al. [27]	69/M	Temporal bone	Well-differentiated	Resection	–	Alive, 24 months follow-up
Macmull et al. [28]	26/M	Femur	Well-differentiated	2 cycles of neoadjuvant MAP, resection, adjuvant 4 cycles ifosfamide & etoposide.	–	Alive, 16 months follow-up
Zhang et al. [29]	26/M	Femur	De-differentiated	Wide resection	–	Alive, 12 months follow-up
Torok et al. [30]	34/M	Femur	Pleomorphic	Wide resection, radiation, chemotherapy	Lung	Died after 16 months, cause unknown
Hamlat et al. [31]	45/F	Thoracic spine	Pleomorphic	Laminectomy T7-T8 and radiotherapy	After 13 months lung and rib	19 months follow-up, gradual deterioration of disease
Torigoe et al. [32]	38/F	Humerus	Pleomorphic	Wide resection with endoprosthesis replacement. Initial high-dose ifosfamide without effect, followed by cisplatin and doxorubicin	Liver	Died after 8 months of disease and liver failure
Lmejjati [33]	45/M	Lumbar spine	Pleomorphic	Emergency decompression at L4/L5 and radiotherapy (45 Gy)	None	Died after 3 months of deterioration from disease
Rasalkar [34]	13/M	Femur	Pleomorphic	Neoadjuvant MAP 2 cycles (methotrexate, adriamycin/cisplatin), Surgery, Adjuvant 1 cycle MAP and further chemotherapy with combinations of ifosfamide/etoposide and adriamycin/cisplatin	Lung	No recurrence of local disease and complete response at 13 months follow-up

In most previous reports, plain radiographs of primary liposarcoma of bone have shown an ill-defined osteolytic lesion, but sclerotic areas have also been noted [4, 21, 22, 25, 32]. In this case, the tumour contained spiculated areas of ossification with evidence of cortical destruction and a soft tissue mass. These features suggested osteoid/bone formation and the favored radiological diagnosis pre-biopsy was osteosarcoma. MRI showed that the lesion was of heterogeneous isointense signal on T1- and high signal on T2-weighted sequences respectively. Areas of high T1 signal suppressed with fat saturation, supporting a partly fatty matrix. Heterogeneous hyper-intensity on T1 and T2-weighted images has been described in previous case reports [32, 34]. Haemorrhage could account for high T1 and T2 signal areas but, in our case methaemoglobin did not suppress with fat saturation.

Our case showed typical histological features of pleomorphic liposarcoma. There were numerous small and large lipoblasts as well as many large tumour cells with vacuolated cytoplasm and highly pleomorphic nuclei; in addition, there were many brown fat-like cells with atypical large hyperchromatic nuclei. Tumour cells strongly expressed FABP4/aP2, a marker of adipose differentiation [6], and UCP1, a marker of brown fat cells [8–10]. FABP4/aP2 has consistently been shown to be expressed in soft tissue tumours of adipose differentiation and is useful in distinguishing primary pleomorphic liposarcoma from other soft tissue pleomorphic sarcomas [6]. FABP4/aP2 is also expressed by brown and white fat cells in hibernoma of bone [7]. In our case, expression of FABP4/aP2 was useful in confirming the morphological diagnosis of primary pleomorphic liposarcoma and excluding osteosarcoma and un-differentiated pleomorphic sarcoma, both of which are FABP4/aP2 negative [6]. Expression of UCP1 was consistent with the finding of numerous brown fat–like cells within the tumour. UCP1 was also expressed by lipoblasts in the tumour. Our case did not show tumour cell expression of S100 or evidence of MDM2/CDK4 amplification. Absence of S100 has been noted in more than 50% of soft tissue pleomorphic liposarcomas in which MDM2 and CDK4 amplification is also typically absent [35].

Most previously reported intraosseous liposarcomas have been noted to exhibit morphological features in keeping with the pleomorphic liposarcoma subtype. However, other high-grade primary malignant tumours containing liposarcomatous elements have been described in bone. These include a liposarcoma containing osteosarcomatous foci [25], a mesenchymoma of bone showing liposarcomatous features [36] and a primary dedifferentiated liposarcoma of the femur that presented as a malignant fibrous histiocytoma [29]. Downey et al. [25] specified that several criteria must be met for the acceptance of a diagnosis of a primary intraosseous liposarcoma of bone. First, it must be proved that the tumour has arisen primarily within the bone i.e. that it is not a metastatic deposit and that it is not periosteal in origin, involving the cortex and marrow secondarily. In addition, a predominance of immature pleomorphic, often bizarre, uni-globular and multi-globular lipoblasts should be noted histologically. Our case meets these criteria, having arisen within the tibia and containing numerous vacuolated tumor cells including lipoblasts. It should be noted that the WHO definition of liposarcoma of bone does not exclude origin from the bone surface [37]; it specifies that the tumour is “a malignant neoplasm whose phenotype recapitulates fat and arises within or on the bone surface.” Based on the findings in the present case, we propose that immunophenotypic expression of markers of adipocyte differentiation (i.e. FABP4/aP2 and UCP1) could usefully be added to the list of criteria for the diagnosis of primary liposarcoma of bone.

Outcome following surgery alone of primary liposarcoma of bone is relatively poor, and there is little data on the use of adjuvant or neoadjuvant chemotherapy or radiotherapy. A review of the literature shows that 16 patients (55%) died after a mean of 13 (range, ½–36) months, whereas 11 (38%) were alive at a mean follow-up of 28 (range 5–60) months (Table 1). Only five cases were treated with chemotherapy. In one case neoadjuvant MAP chemotherapy resulted in 54% tumour necrosis; after surgery the treatment was switched to ifosfamide and etoposide with no recurrence reported after 13 months follow-up [18]. Torigoe et al. reported no effect of high dose ifosfamide; a change to cisplatin and doxorubicin resulted in liver toxicity and deterioration [32]. Macmull et al. [28] found no effect with doxorubicin, and when chemotherapy was changed to ifosfamide and etoposide no relapse after 16 months follow up. In a further two cases treated with chemotherapy, there was no reported efficacy, both patients dying within 1.5 years of follow-up [19, 20]. There is now discussion within the sarcoma community that rare high-grade primary malignant bone tumours should have their chemotherapy tailored according to their individual histological subtype [38, 39]. In our case neoadjuvant MAP treatment resulted in an unexpectedly good tumour response after two cycles, with background FDG signal of the metastatic sites of disease and more than 95% necrosis in the primary tumor noted after surgery. Pleomorphic liposarcoma of bone is rare tumour and there is no clinical trial data available to guide the optimal chemotherapeutic approach. Treatment findings in our case provide some support for the use for neoadjuvant MAP chemotherapy to treat this rare tumour.
